# Early open reduction of dislocated hips using a modified Smith-Petersen approach in arthrogyposis multiplex congenita

**DOI:** 10.1186/s12891-020-3173-0

**Published:** 2020-03-04

**Authors:** Mingyuan Miao, Haiqing Cai, Zhigang Wang, Liwei Hu, Jingxia Bian, Haoqi Cai

**Affiliations:** 1 Department of Orthopedic Surgery, Shanghai Children’s Medical Center, Shanghai Jiao Tong University School of Medicine, Shanghai, China; 2 Department of Radiology, Shanghai Children’s Medical Center, Shanghai Jiao Tong University School of Medicine, Shanghai, China

**Keywords:** Arthrogryposis, Hip dislocation, Open reduction, Modified Smith-Petersen approach, Preserved rectus femoris

## Abstract

**Background:**

Arthrogryposis multiplex congenita (AMC) is a rare syndrome with multiple joint contractures. Within the medical community, there is controversy surrounding AMC in terms of the ideal surgical approach and age for performing a reduction of dislocated hips. The purpose of this retrospective study was to evaluate the clinical outcomes of early open reduction of infant hip dislocation with arthrogryposis multiplex congenita following a modified Smith-Petersen approach that preserves the rectus femoris.

**Methods:**

From 2010 to 2017, we performed this procedure on 28 dislocated hips in 20 infants under 12 months of age with AMC. The clinical and radiology data were reviewed retrospectively. The mean age at surgery was 6.9 ± 5.1 months, with a mean follow-up of 42.4 ± 41.1 months.

**Results:**

After open reduction, the average hip acetabular index (AI), the international hip dysplasia institute classification (IHDI), and the hip range of motion significantly improved (all *P* < 0.001). After the surgery, 16 patients were community walkers, and four patients were home walkers. Three hips in two patients required secondary revision surgery for residual acetabular dysplasia with combined pelvic osteotomy and femoral osteotomy. Seven of the hips that had been operated on showed signs of avascular necrosis (AVN). Among them, four were degree II, two were degree III, and one was degree IV. Multiple linear regression analysis demonstrated that greater age (in months) heightened the risk for secondary revision surgery (*P* = 0.032).

**Conclusions:**

The modified Smith-Petersen approach preserving the rectus femoris is an encouraging and safe option for treating hip dislocation in young AMC patients (before 12 months). If surgery takes place at less than 12 months of age for patients with AMC, this earlier open reduction for hip dislocation may reduce the chances of secondary revision surgery.

**Level of evidence:**

IV, retrospective non-randomized study.

## Background

Arthrogryposis multiplex congenita (AMC) is a rare disorder characterized by congenital, non-progressive, and multiple joint contractures with replacement of the musculature with fibrous bands and fat tissue. AMC is not a homogenous disease, with more than 150 recognized conditions involving arthrogryposis [1].

The most widely accepted classification of AMC was first proposed by Bamshad [2]. AMC with normal neurological functioning is divided into two subtypes: amyoplasia and distal arthrogryposis. Amyoplasia is the most common of these subtypes and occurs in 38–43% of AMC cases [3, 4].

Hips affected by AMC typically are abducted, flexed, and externally rotated [5]. Hip deformity in AMC is defined as a teratological dislocated hip (TDH) condition, which is not reducible by gentle manipulation at birth. Hip dislocation is present in approximately 30% of patients with amyoplasia-type AMC [5]. The acetabulum is shallow and small, and the femoral head is hypoplastic and often flattened in its medial part. The anteversion angle may vary greatly and can even be retroverted [6]. These pathological features cause difficulties in dealing with TDH in AMC.

The previous literature demonstrates significant inconsistency regarding the appropriate surgical approach and age for performing reduction of dislocated hips in AMC [7]. Closed reduction results generally have been considered poor, often causing increased stiffness and redislocation. On the other hand, open reduction is also controversial, especially in cases of bilateral dislocation and increased surgery age. Gruel et al. recommended against reducing bilateral TDH because of high rates of resulting redislocation, subluxation, and avascular necrosis [8]; meanwhile, Bernstein et al. argued that open reduction should be performed between 6 months and 1 year of age [9]. Bahattin et al. held yet another perspective, arguing that short-term results of early (before 6 months) open reduction were not promising, given that about half of cases receiving early surgery required additional hip surgeries [7]. Wada reported that femoral varus derotation osteotomy and pelvic osteotomy were combined synchronously in most open reductions [10]. Although most surgeons select the anterolateral approach (Smith-Petersen approach) for reduction, some studies report better clinical results following the medial approach (Ludloff’s medial approach) [9]. Most research regarding open reduction through the Smith-Petersen approach has focused on children over 12 months old [10–12].

Based on prior studies, this study hypothesized that a modified Smith-Petersen approach would be effective for AMC and that there would be a negative correlation between reduced age and surgical outcome.

## Methods

This retrospective study was approved by the Medical Ethical Committee of Shanghai Jiao Tong University School of Medicine and the affiliated Shanghai Children’s Medical Center. Written informed consent was obtained from the legal guardian of each participant in the study. AMC was diagnosed in accordance with Fisher’s clinical criteria [13]. Hip dislocations were diagnosed by pelvis X-ray and physical examination. The patients included in this study were AMC patients with TDH who were treated at the same hospital by senior pediatric orthopedic surgeons between 2010 and 2017. Inclusion criteria included being less than 12 months old at the time of hip reduction and having at least 24 months of follow-up care after the open reduction. None of the patients included had undergone previous treatment. Physical and roentgenographic examinations were performed in outpatient follow-up.

### Physical examination

As previously described, the degree of hip functioning was not the only criterion for classifying the results. All the patients also were classified as community walkers, home walkers, non-functional walkers, or non-walkers in the final post-operative follow-up [6, 14]. The preoperative and postoperative clinical evaluations measured the degree of joint mobility. The hip range of motion in flexion and abduction also was assessed at the last follow-up [6].

### Radiological assessment

The IHDI classification, continuity of Shenton’s line, AI, ossific nucleus center edge angle (ONCEA), and signs of AVN were evaluated in the final follow-up [15–17]. AVN also was classified in accordance with Kalamchi-MacEwen classification [18]. Hips were considered to be centered if the Shenton arc was continuous, and the CE angle was 15° or greater. For patients who had bilateral TDH, both hips were reduced in the same surgical treatment.

### Surgical treatment

**Fig. 1:**
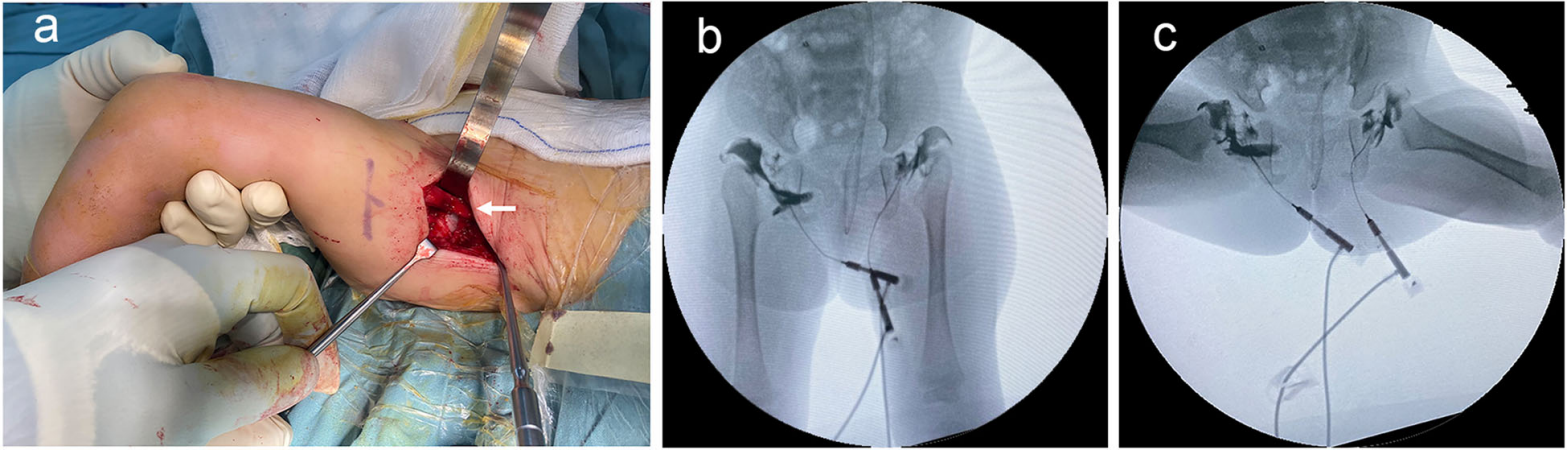
**a** Photos during operation indicated the straight heads of rectus femoris were not released (the white solid arrow) and exposed the femoral head after opening the hip joint capsule through T-shaped incision. **b** and **c** Preoperative arthrography shows the hip inverted limbus with widened acetabulum medial pooling in AMC. Tentative closed reduction shows that the dislocated hips were stiff and irreducible

All of the hips were operated on using a modified Smith-Petersen approach, but the rectus femoris was preserved through an incision below the iliac crest [19, 20]. The iliac apophysis was split and the iliac wing exposed subperiosteally. The straight heads of the rectus femoris were not released, but that of the iliopsoas muscle was released. Meanwhile, the tendon attachment of iliopsoas muscle preserved (Fig. 1a). A T-shaped incision was made from the most medial aspect of the hip capsule to the most lateral. The soft tissue was removed, including the ligamentum teres in the acetabulum cavity. The transverse ligament also was divided. The femoral head was gently reduced without tension, and then the hip was moved through a complete range of motion to determine the stable reduction position. Open reductions were followed by the application of a hip spica cast in the human position with a gentle posterior mold at the greater trochanteric region of the femur. Adductor tenotomy was done in all hips to reduce tension and to achieve better congruity [10].

The patients were immobilized after surgery using a hip spica cast in the human position for a period of 12 weeks, after which they wore a brace for 6 months.

### Statistical analyses

Numerical variables are shown as mean ± standard deviation. The results were analyzed using an independent-sample t-test and paired t-test. Multiple linear regression models were performed to evaluate the relationships between secondary revision surgery and related factors. The association between the revision osteotomy surgery and related factors—including sex, surgery age (months), preoperative AI, and preoperative IHDI classification—was evaluated using logistic regression models to determine the odds ratio (OR). Multivariate-statistical significance was assumed at *P* < 0.05, *P* < 0.01, and *P* < 0.001. All analyses were performed using a statistical software package (SPSS for Windows, v. 20.0; SPSS, Inc., Chicago, IL).

## Results

### Baseline data

Among the 20 patients who participated in this study, seven were male, and 13 were female. Eight patients presented with bilateral TDH, and 12 had unilateral dislocation (Table 1). The children’s mean age at the time of surgery was 6.9 ± 5.1 months (range: four to 12 months). The mean duration of the follow-up was 42.4 ± 41.1 months (range: 25 to 99 months).

**Table 1:** Characteristics of AMC patients with TDH and prognostic factors for treatment outcome in the revision group

	All	Revision	Non-revision	*P* value*	OR
Numbers (patients/hips)	20/28	2/3	18/25		
Side (left/right)	14/14	1/2	13/12		
Sex (male/female)	7/13	0/2	7/11	0.324	
Open reduction age (months)	6. 9 ± 5.1	10 ± 1.7	6.5 ± 2.4	0.032	4.588
Preoperative AI	39.9 ± 4.4	38.8 ± 5.4	40.1 ± 4.4	0.07	
Preoperative IHDI (II/III/IV)	1/19/8	0/2/1	1/17/7	0.913	

*represents statistical differences (*P* < 0.05). *OR* Odds ratio

### Ambulatory and hip function status

In relation to ambulatory function, 16 patients were community walkers, and four were home walkers. Thirteen patients were able to walk without aids and seven were able to walk with braces.

Before open reduction, the mean hip range of motion in flexion and extension in the postoperative examination was 78.2 ± 15.2° (range: 50° to 105°), and the mean range of motion in abduction was 23.0 ± 8.6° (range: 10° to 35°). The mean hip range of motion in flexion and extension in the postoperative examination was 102.0 ± 16.2° (range: 65° to 125°), and the mean range of motion in abduction was 37.1 ± 9.7° (range: 15° to 50°). Hip flexion/extension and abduction range improved after surgery at a statistically significant level (*P* < 0.001 for both factors).

### Radiologic evaluation

Regarding radiographic evaluations, before open reduction, one hip was IHDI II degree, 19 hips were IHDI III degree, and eight hips were IHDI IV degree. The mean AI value was 39.9 ± 4.4° (range: 30.9° to 48.3°) in the preoperative examination.

**Fig. 2:**
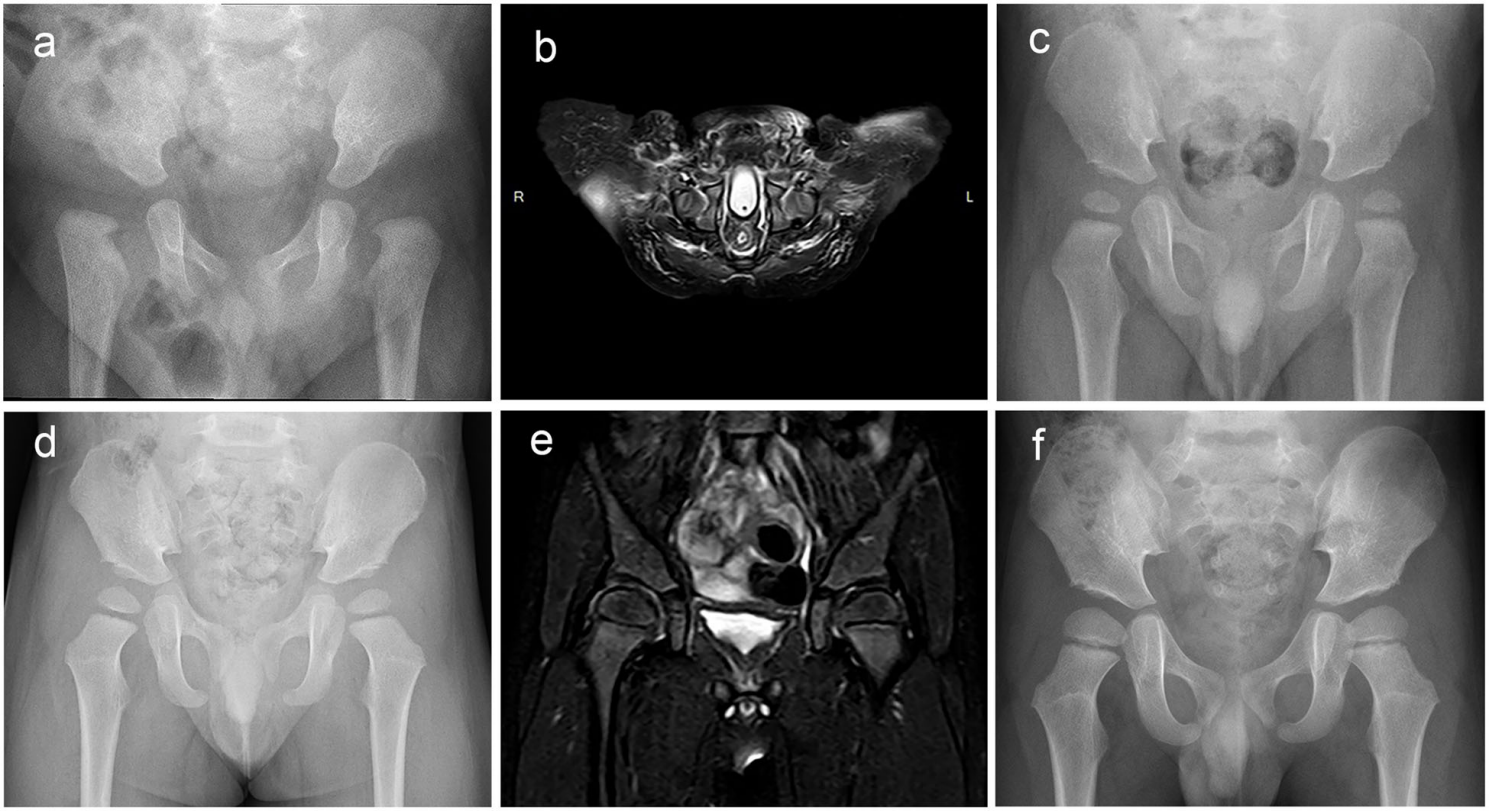
**a** Preoperative pelvic anteroposterior view in a five months old patient. **b** Immediate MRI after surgery confirmed satisfactory hip reduction. **c** and **d** Radiograph at 16 months and 26 months after open reduction, respectively. **e** MRI assessment at 55 months after open reduction. **f** Radiograph at 55 months after open reduction

After open reduction, 25 hips were classified IHDI I degree, and three hips were IHDI II degree. The mean AI was 22.5 ± 5.1° (range: 13.6° to 31.9°) in 28 hips. The mean IHDI classification and AI degree were significantly reduced after surgery, and there were statistical differences (*P* < 0.001 in both cases). The mean ONCEA was 17.1° (range: 9.4° to 30.4°) in 27 hips with apparent ossification in the center of the femoral head. The Shenton’s line was found to be intact for all patients in the final follow-up (Fig. 2). Seven hips showed signs of avascular necrosis. Among them, four were degree II, two were degree III, and one was degree IV.

### Complications and additional procedures

Among the 28 hips, there was no redislocation, but there was one subluxation. Three hips in two children required a pelvic and femoral osteotomy with varus and rotation for residual acetabular dysplasia around the age of 3.4 years. There were two Salter and one Dega osteotomy in pelvic procedures. Multiple linear regression models were performed to evaluate the relationships between revision osteotomy surgery and related factors, including sex, surgery age, preoperative AI, and preoperative IHDI classification (Table 1). There was a significant difference in surgical age between the secondary revision surgery patients and the non-revision surgery patients (*P* = 0.032). The logistic regression model analysis showed that the OR value of open reduction age for the revision groups and non-revision groups was 4.588 (Table 1). The results indicate that age at hip open reduction is significantly associated with secondary revision surgery.

Apart from one subluxation, there were no serious complications (e.g., anesthesia accident, compartment syndrome, hip infection, osteomyelitis). However, there were four superficial wound infections. Table 2 summarizes the additional procedures in 14 patients.

**Table 2:** Additional procedures outside the hip joint in 14 patients

Operative site	Numbers/Surgical procedure
Knee	1/Correction of congenital dislocation of patella
	2/Reconstruction of anterior cruciate ligament and posterior cruciate ligament
	2/Quadriceplasty
	2/Flap surgery and external fixation for knee flexion
Shank	2/Reconstruction of dysplasia of fibula
Foot	4/Astragalectomy
	6/Vertical talus reduction and Kirschner wire fixation
	12/Achilles tendon lengthening
	2/Release through internal posterior approach of foot
Elbow	2/Posterior elbow release
Wrist	2/Wrist extension osteotomy

## Discussion

There is little prior research on treating hip dislocation following the Smith-Petersen approach in AMC for infants. Our study addresses this research gap, as it presents an experience in treating TDH in AMC using the aforementioned approach for children under 12 months old.

The TDH in AMC are much more rigid and irreducible than developmental dysplasia of the hip. Therefore, hip closed reduction generally results in increased stiffness and a high rate of subluxation and redislocation, reducing its favor among surgeons [21]. Open reduction for TDH was introduced for patients with AMC as a valid option due to its ability to prevent pelvic obliquity, sitting imbalance, gait abnormality, and secondary scoliosis [10].

Concerning surgical age, Bahattin et al. argue that early reduction (before 6 months) in AMC does not reduce future hip surgeries [7]. Several authors have suggested that operative treatment of TDH in AMC can be performed at three to 10 months [3, 22, 23]. Hip development involves an intricate balance between changes in the acetabulum and the proximal femur [24]. Considering the severe deformity of TDH and the corresponding need for earlier and longer acetabulum molding, we agree with their treatment window at three to 10 months. In fact, the results in this study confirm this viewpoint. The two patients (three hips) requiring secondary revision surgery were more than 8 months old when they received open reduction; however, 15 out of the total 18 participants did not require secondary revision surgery at less than 8 months old. There was a significant difference in the age of surgery between the secondary revision surgery patients and the non-revision surgery patients. However, we sometimes did not receive the patients within the ideal timeframe because they were abandoned by their families and sent to welfare institutions. In the literature, a relatively high proportion of the reported AMC case series required additional femoral or pelvic osteotomies along with open reduction. These results suggest that the effects of reduction age on acetabular shaping in TDH and developmental hip dysplasia are similar; in other words, reduction surgery at an older age is more likely to lead to residual acetabular deformities [25].

We used a modified anterior Smith-Petersen approach that preserved the rectus femoris for all the TDH in this research. There are several advantages to this method. First, it allows more attention to be paid to concentric reduction and reduction stability in TDH in AMC. The acetabulum in AMC is small, shallow, and filled with fibrous-fatty tissue. The femoral head in AMC is hypoplastic and often flattened in its medial portion, which can be demonstrated by arthrography (Fig. 1b and c) [6]. Compared with medial-approach open reduction, the anterior Smith-Petersen approach is demonstrably more effective in hip exposure, obstacle removal, and circumferential capsulotomy. The reports in the literature show that there are more frequent secondary procedures for progressive subluxation after open reduction when using the anteromedial access route [3, 8]. Additionally, the medial approach is likely related to injury of the medial circumflex artery, which causes iatrogenic AVN, especially in infants younger than 12 months with unclear hierarchical anatomy. The reported rate of significant AVN in medial-approach open reduction is as high as 43% [26, 27]. In the present research, we preserved the rectus femoris and the tendon attachment of iliopsoas in this modified anterior Smith-Petersen approach, thus minimizing muscle damage near the hip.

Avascular necrosis of the femoral head in open reduction through the Smith-Petersen approach in TDH is a risk when doing a complete capsulotomy of the hip. However, Akazawa et al. indicated that hip capsulotomy adjacent to the acetabular rim would not affect the blood supply to the femoral head if the incision is sufficiently distant from the base of the femoral neck. The lateral epiphyseal artery comes from the femur greater trochanter and passes through the posterior capsule at the femoral neck base [10, 12]. In our series, seven hips demonstrate AVN, but only one hip showed the Kalamchi and MacEwen grade IV AVN.

Open reduction often has been associated with increased stiffness of the hip in AMC [21]. In terms of the open reduction approach, Staheli et al. reported that the range of motion of AMC patients who received the medial approach was better than that of those treated using an anterolateral approach [23]. We believe that the limited hip motion is related to the added soft tissue injury that occurs with the anterolateral approach. Therefore, we have preserved the rectus femoris and the attachment of iliopsoas in our Smith-Petersen approach, which also maximizes postoperative hip function. In our series, most of the children retained a certain degree of hip joint activity, and none of them encountered joint stiffness after the operation.

Bahattin et al. suggested that open reduction for TDH at a late age may be preferable because open reduction and femoral osteotomy procedures can be performed simultaneously to reduce the need for additional surgeries [7]. However, when the femora-acetabular harmony is created early through the open reduction, the remodeling capacity of the femoral head and the acetabulum can be maximized, reducing the need for additional surgeries. In our study, 25 hips were IHDI I degree, and three hips were IHDI II degree, without IHDI III or IV classification postoperatively. Only three hips received secondary revision surgery including femoral and pelvic osteotomy in the latest follow-up. These results indicate favorable results for femora-acetabular harmony after early open reduction through a modified Smith-Petersen approach that preserves the rectus femoris.

The reduction of bilateral TDH in AMC remains controversial. Many authors have argued that bilateral TDH should be left untreated because the pelvis remains level and motion is satisfactory; leaving it untreated also circumvents the high rate of complications after surgery [1]. Some authors suggest that bilateral TDH should be reduced to restore femora-acetabular harmony and decrease the risk of later pain or stiffness [3, 12]. In our study group, eight patients with bilateral TDH received open reduction surgery simultaneously, and seven of these patients did not require secondary revision surgery. There was no statistically significant difference in surgical age and revision surgery between the bilateral and unilateral hip groups (*P* = 0.188 and *P* = 0.736). These results may suggest that simultaneous open reduction of bilateral hip joints has little effect on clinical results.

TDH in AMC is accompanied by multiple musculoskeletal disorders. Management of arthrogryposis is difficult because numerous surgical procedures are necessary for concomitant knee, shank, foot, elbow, and wrist deformities [10]. We dealt with lower limb deformities in the following order: foot, hip joint, and knee joint.

Our study had several limitations. First, because it was a retrospective study, there was selective bias and no standardized indication for secondary revision surgery. Secondly, the AMC sample size was too small. Having a larger number of patients in future research may yield more definitive results concerning the best age-related opportunity for early open reduction surgery. Thirdly, we only evaluated medium-term clinical outcomes. Longer follow-up may lead to increased incidence of secondary revision surgery and complications; it may also provide additional clinical information.

## Conclusion

Our study indicates that early open reduction of dislocated hips through a modified Smith-Petersen approach preserving the rectus femoris in AMC could restore femora-acetabular harmony and provide sufficient clinical and radiological results. Earlier open reduction for hip dislocation may reduce the chances of secondary revision surgery.

## Data Availability

The datasets generated and/or analyzed during the current study are not publicly available because they contain patients’ personal information, but they are available from the corresponding author upon reasonable request.

## Abbreviations

AI: Acetabular index
AMC: Arthrogryposis multiplex congenita
AVN: Avascular necrosis
IHDI: The international hip dysplasia institute classification
OR: Odds ratio
TDH: Teratological hip dislocation
